# Uncommon Surgical Management by AVF between the Great Saphenous Vein and Anterior Tibial Artery for Old Radiocephalic AVF Failure

**DOI:** 10.3390/life12040529

**Published:** 2022-04-03

**Authors:** Réka Kaller, Adrian Vasile Mureșan, Emil Marian Arbănași, Eliza Mihaela Arbănași, István Kovács, Emőke Horváth, Bogdan Andrei Suciu, Ioan Hosu, Eliza Russu

**Affiliations:** 1 Clinic of Vascular Surgery, Mures County Emergency Hospital, 540136 Targu Mures, Romania; reka.kaller@umfst.ro (R.K.); adrian.muresan@umfst.ro (A.V.M.); eliza.russu@umfst.ro (E.R.); 2 Department of Surgery, George Emil Palade University of Medicine, Pharmacy, Science, and Technology of Targu Mures, 540139 Targu Mures, Romania; bogdan.suciu@umfst.ro; 3 Faculty of Pharmacy, George Emil Palade University of Medicine, Pharmacy, Science, and Technology of Targu Mures, 540139 Targu Mures, Romania; arbanasi.eliza-mihaela@stud18.umfst.ro; 4 Clinic of Cardiology, Mures County Emergency Hospital, 540136 Targu Mures, Romania; istvan.kovacs@umfst.ro; 5 Department of Pathology, George Emil Palade University of Medicine, Pharmacy, Science, and Technology of Targu Mures, 540139 Targu Mures, Romania; emoke.horvath@umfst.ro; 6 Department of Anatomy, George Emil Palade University of Medicine, Pharmacy, Science, and Technology of Targu Mures, 540139 Targu Mures, Romania; 7 Department of Nephrology, Mures County Emergency Hospital, 540136 Targu Mures, Romania; ioan.hosu@umfst.ro

**Keywords:** arteriovenous fistula, lower extremity fistula, hemodialysis, fistula complication, rupture, hemorrhage

## Abstract

Introduction: Autologous native arteriovenous fistula (AVF) created in the non-dominant arm is the gold standard vascular access for dialysis in end-stage renal disease, but the post-surgical vascular access dysfunction causes a reduction in the patient’s quality of life. Creating a functional upper extremity permanent arteriovenous access is limited by the upper limb’s vascular resources, so good management of a complicated arteriovenous fistula may improve patient outcomes. This article highlights the importance of new surgical options in treating complicated AVFs. Case report: We present the case of a patient with a 17-year-old complex radio-cephalic arterio-venous fistula and a series of surgical interventions performed for life salvage in the first place and functional vascular access in the second place. Furthermore, we describe a successfully created uncommon type of fistula in the lower extremity between the great saphenous vein and the anterior tibial artery as the last possible access for hemodialysis in this patient. Results: The patient underwent the first successful dialysis using the newly created lower limb fistula 1 month after the surgery. Conclusion: Applying new surgical techniques to manage AVFs gives a unique chance to improve the quality of life and reduce morbidity and mortality in these patients.

## 1. Introduction

Using data from Romanian Renal Registry, the number of dialysis patients exceeded 13,400, of whom 236 patients were using peritoneal dialysis. In 2021, the number of newly admitted patients up until the present day was 2754, with a slightly decreasing tendency [1]. The prognosis for 2022–2023 shows an expected disappearance of peritoneal dialysis.

Comparing the data with the European Registry, the incidence of dialysis initialization was higher in 2019, putting Romania in tenth place among all European countries [1].

Dysfunction of vascular access has remained one of the leading causes of aggravation of the disease and increase in mortality in hemodialysis patients; in this context, a functional arteriovenous fistula remains the dialysis patient’s lifeline [2].

The upper limb arterio-venous fistula (AVF) is well described in the literature. However, ankle AVF is not a common site. Still, there are some papers describing vascular access in the lower limb using the posterior tibial artery and the great saphenous vein [3,4].

The repair of a long-term functional and complicated AVF is always tricky and is a big challenge for the surgeon, but it is worth a try. This paper aims to present the importance of surgical treatment of 17 years’ functional but ruptured radio-cephalic fistula and afterward a successful creation of a new arteriovenous fistula between the great saphenous vein and the anterior tibial artery in a patient left without any other vascular access possibilities except a life-long catheter.

## 2. Case Report

**Figure 1:**
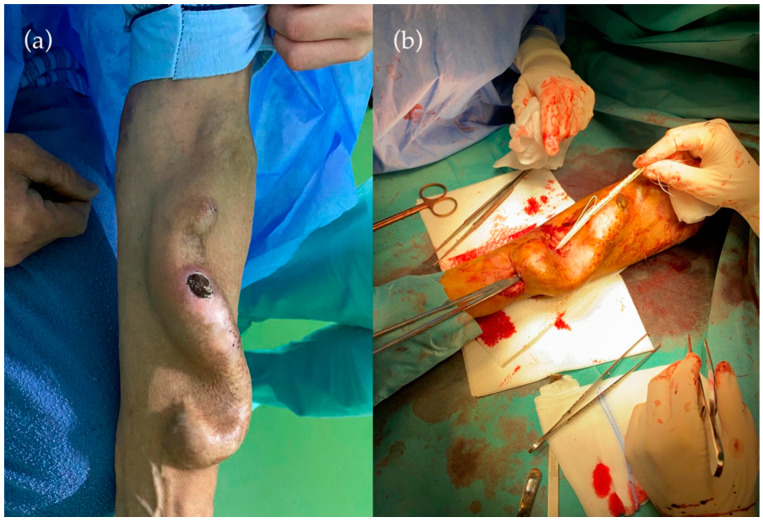
(**a**) Radio-cephalic fistula aneurysmatic dilated with the presence of an area of necrosis in the cephalic vein. (**b**) Fistula ligation immediately after anastomosis using a Dacron-type circular prosthesis.

We focused on a 54-year-old male patient known to have stage 5 KDOQI chronic kidney disease on hemodialysis, unilateral renal agenesis, right renal artery stenosis, and secondary hypertension, present with a 17-year-old functional but aneurysmatic dilated, calcified left radio-cephalic fistula, with an approximately 1 x 1 cm skin necrosis at the level of the vein. As the first surgical approach, ligation of the fistula using a circular section of a Dacron prosthesis as a cerclage and a new brachiocephalic fistula in the left arm were performed (Figure 1).

This new fistula was immediately used for dialysis. This approach was chosen for technical reasons as the cephalic vein was heavily calcified, rendering a cut through it and then suture impossible. Therefore, closing the fistula was merely prophylactic to avoid cardiac insufficiency onset and the disruption of the fistula until the definitive solution of completely resecting the calcified vein, followed by the performing of a new fistula.

**Figure 2:**
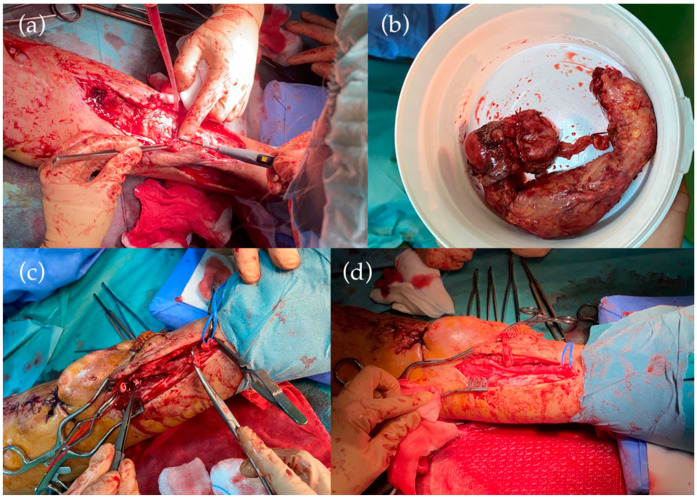
(**a**) Resection of the aneurysmatic dilated cephalic vein. (**b**) Cephalic vein dilated aneurysmatic, intensely infiltrated atherosclerotic. (**c**) Resection of the radial artery at a distance of 4 cm, at the level of the arteriovenous anastomosis. (**d**) Reconstruction of the radial artery with interposition of the left internal saphenous vein inverted.

Before the planned surgical intervention, the patient was presented to the Emergency Room with active bleeding in the left forearm and edema of the left half of the body. A Computed Tomography angiography (CTA) described a left central brachiocephalic vein thrombosis. The surgical exploration showed the recanalization of the ligated radiocephalic fistula and rupture at the level of the skin ulceration. As an emergency intervention, the patient underwent a second ligation and suture of the ruptured fistula for life salvage. Despite the life-saving operation, the fundamental problem was not solved; further interventions were necessary (Figure 2).

Complete resection of the ruptured fistula, including a small part of the radial artery at the level of the anastomosis, was performed. A short radial interposition using the great saphenous vein harvested from the left lower limb ended the intervention. In addition, the ligation of the newly created left brachiocephalic fistula became mandatory because of venous hypertension caused by the left central brachiocephalic vein thrombosis. Two reinterventions were needed due to hemorrhagic complications.

The histopathological examination of the excised vein confirmed heavy calcification within the atherosclerotic modifications.

Completing the patient’s journey, a forearm skin reconstruction with a split-thickness skin graft (STSG) was performed. The patient’s hemodialysis was managed using a temporary tunneled catheter in the left femoral vein during all this time.

One month after discharge, a new radiocephalic fistula was performed in the right forearm, which would also be closed later because of symptomatic ischemic steal syndrome. Atherosclerosis-affecting antebrachial arteries led the surgeons not to ligate the radial artery distal to the anastomosis.

**Figure 3:**
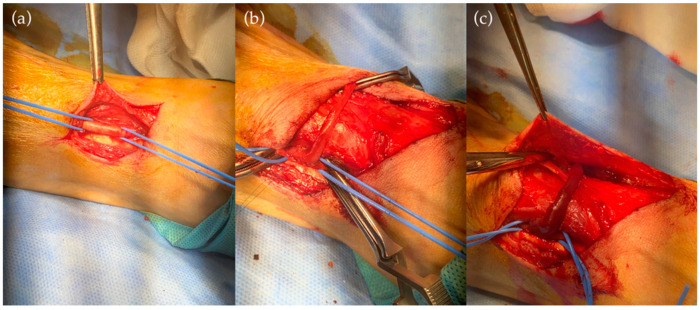
(**a**) Preparation and suspension on cords of the anterior tibial artery. (**b**) Anastomosis between the right internal saphenous vein and the right anterior tibial artery. (**c**) Anastomosis patent post-decamping.

After multiple surgical procedures involving both upper extremities to reestablish functional vascular access for dialysis, it was decided to try, as the last possibility, an arteriovenous fistula in the lower limb. After ultrasound mapping of the right lower limb vessels, the vascular surgeons performed a fistula between the great saphenous vein and the anterior tibial artery with the aid of a sciatic lumbar plexus block. The ligation of all of the collateral branches of the vein was performed. Due to massive atherosclerosis, the shunt between the saphenous vein and the posterior tibial artery (preferred in this situation) was not possible. The procedure had no hemorrhagic complications (Figure 3).

After 4 weeks, the right lower limb became critically ischemic. In the absence of other hemodynamical explanations, peripheral angiography was decided. Several serial critical stenoses of the posterior tibial artery were identified and managed by balloon dilatations and stent implantation at the distal level of the posterior tibial artery. The angiographical result, as well as the clinical evolution, was good.

## 3. Results

**Figure 4:**
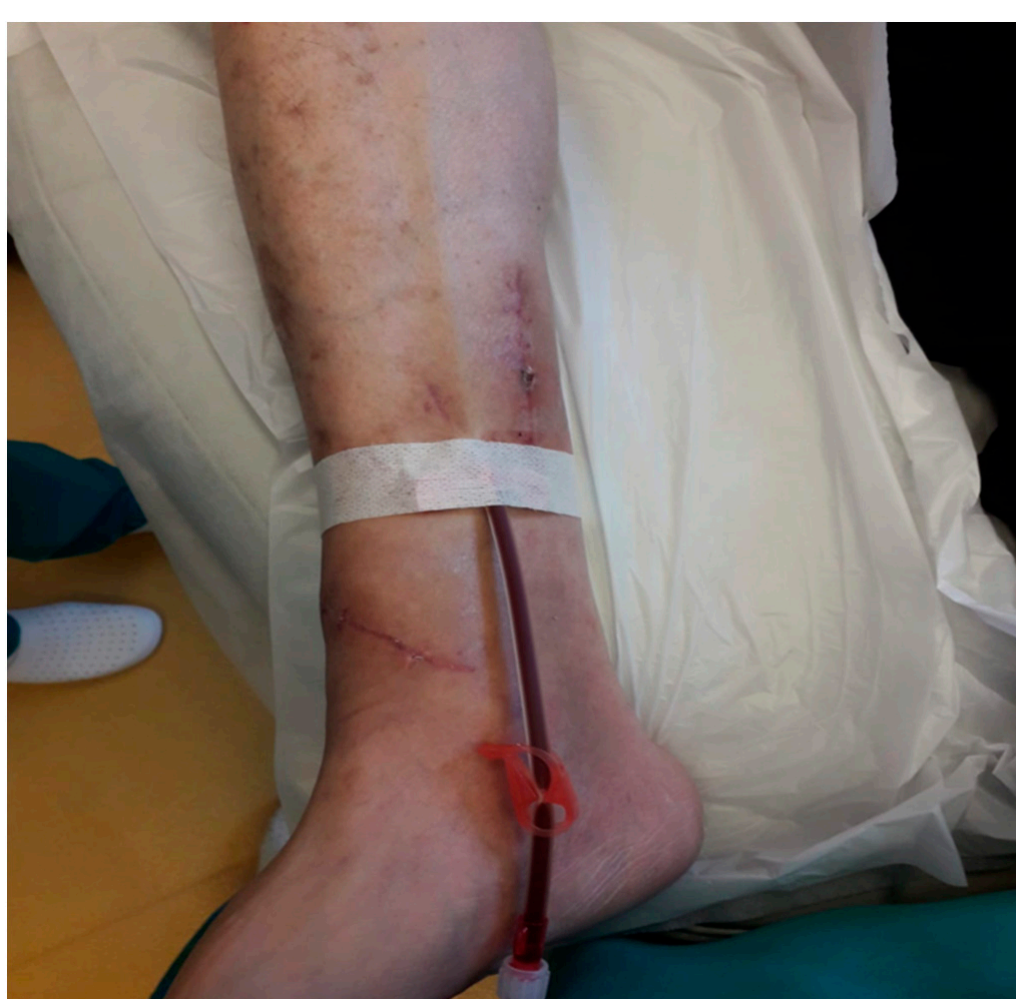
Initiation of dialysis in the right tibial-saphenous arteriovenous fistula.

On the seventh postoperative day, the patient was discharged with a functional fistula in the right lower limb. One month later, the patient underwent the first successful hemodialysis on the lower limb fistula (Figure 4).

Follow ups after 3 months and 6 months acknowledged the satisfactory performance of the lower leg fistula. Additionally, reconstruction of the left upper limb circulation saved the patient’s life.

## 4. Discussion

The radiocephalic arteriovenous fistula performed at wrist level is the recommended first choice of vascular access for hemodialysis. In comparison with grafts and catheters, the native fistula increases the life expectancy of these patients [2].

After an arteriovenous fistula is created, local hemodynamic changes occur, as the fistula is the result of the connection between a high-pressure artery and a low-pressure vein. The rate of complications increases as the patency rate decreases, as was observed on elderly patients benefiting for long periods from hemodialysis. Additionally, the age of the fistula is directly proportional to the occurrence of fistula failure [5].

Vascular access dysfunction is a global problem and the most important cause of mortality in these patients. Current literature describes the following as the most frequent fistula complications: aneurysm, rupture, thrombosis, stenosis [6], infection, steal syndrome, and venous hypertension [7]. Four of these occurred from these seven types of possible complications in our case. The most critical moment in the management of this case was the rapid gain of functional vascular access after the patient was safe from any hemorrhagic complications. The first technical challenge of the case was the ligation of an old, calcified, dilated fistula [8]. It failed because of recanalization and rupture despite carefully planning the first intervention. The technique of ligating the cephalic vein with a circular section of a Dacron prosthesis as a cerclage was a good decision since massive calcification made simple suture unreliable. Further operations aimed to finally remove the damaged fistula and the revascularization of the upper arm [9].

The Kidney Disease Outcomes Quality Initiative (KDOQI) considers it essential to educate patients in emergencies, such as aneurysm rupture or hemorrhage, and to recognize the symptoms of fistula complications, such as thrombosis, stenosis, or fistula dysfunction [10]. The general practitioner has a vital role in this situation, second in line after the nephrologist. The dialysis patients present an increased risk for cardiovascular morbidity and mortality. For this reason, monitoring these patients is essential to prevent any complications [11].

Unfortunately, either venous resources in the upper limb often become depleted or the vessels at this level do not have the necessary patency to create reliable fistulas. Whenever preservation of the functionality of the upper limb fistulas is impossible, we have to try to develop fistulas in the lower limbs [12]. Despite the little experience in this type of fistula, it is a better option for the patient than a synthetic graft or a central venous catheter [3].

An arteriovenous fistula in the lower limb is not an experimented option, yet the medical literature describes some cases with relatively high patency rates. The most frequently described fistula in the lower limb is between the posterior tibial artery and the great saphenous vein [4]. An arteriovenous fistula between the anterior tibial artery and the great saphenous vein is not the most frequent option. The weakness or risk of this procedure is the steal syndrome caused by atherosclerosis, causing critical limb ischemia. The clinical outcome was essentially better after endovascular stent implantation in the posterior tibial artery [13,14]. Despite the diagnosis of peripheral artery disease, highlighted using preoperative ultrasonographical mapping of the lower limb vessels, we still decided to perform the AVF in the lower limb because we considered this procedure superior to the peritoneal dialysis [15].

The patient’s follow-up was monthly, consisting of ultrasonographic examination, flow measurement, and peak systolic velocity recording [16]. Postoperative ultrasonography is the examination of choice for monitoring the maturation of the AVF and diagnosing the complications [17]. As a thorough follow-up, medical treatment is also essential. A prospective cohort study in 2019 shows that Aspirin has a vital role in preventing AVF dysfunctions [18].

The particularity of this case consists of a complex therapeutical approach and a unique type of arteriovenous fistula in the lower limb. Preoperative angiographic exploration is recommended when this type of fistula is planned. Careful assessment must be made to foresee the possibility of a steal syndrome. As appealing and effective as the ankle approach may look, it should only be performed after ruling out every other access site in the upper limb.

As a final idea, we consider that the most critical aspect of the correct management of a complicated arteriovenous fistula is, first, to improve the quality of life in these patients and to decrease the morbidity and mortality and, second, to develop new dialysis techniques that are cost-effective, accessible, and offer good outcomes [18].

## 5. Conclusions

The surgical management of the complicated arterio-venous fistulas is essential for decreasing the morbidity and mortality of hemodialysis patients. We consider the particularity of the presented case is the authenticity of the surgical techniques, which are not commonly described in the literature. We also want to emphasize the importance of an arterio-venous fistula in the lower limb, which is not a frequently reported option but is an important one in the long-run benefit of improving the patient’s quality of life. Additionally, an angiography is highly recommended to avoid critical limb ischemia before this procedure.
